# Influence of side-hole configuration on recirculation in dual-lumen cannulas for veno-venous extracorporeal membrane oxygenation: a parametric computational fluid dynamics study

**DOI:** 10.3389/fbioe.2026.1911749

**Published:** 2026-09-18

**Authors:** Chenying Zhu, Ping Ye, Zhaohua Chang

**Affiliations:** School of Health Science and Engineering, University of Shanghai for Science and Technology, Shanghai, China

**Keywords:** CFD, dual-lumen cannula, recirculation, shear stress, VV-ECMO

## Abstract

**Introduction:**

Veno-venous extracorporeal membrane oxygenation (VV-ECMO) increasingly utilizes dual-lumen cannulas (DLCs) to facilitate single-site cannulation. However, challenges such as recirculation and elevated wall shear stress (WSS) continue to impede their efficacy. The influence of the geometric configuration of drainage side holes on these hemodynamic parameters remains inadequately characterized.

**Methods:**

In this study, we performed transient, non-Newtonian computational fluid dynamics (CFD) simulations on eight distinct DLC configurations subjected to pulsatile venous inflow. These simulations were conducted within an idealized model of the vena cava and right atrium (RA), developed in accordance with ISO 18193:2021 standards. The study systematically varied the axial spacing between drainage holes and the infusion port (denoted as H_1_/H_2_), as well as the circumferential and axial arrangement of side holes in the superior vena cava (SVC) and inferior vena cava (IVC).

**Results:**

Results indicate that the recirculation fraction (RF) is influenced by two geometric factors. First, among six configurations with fixed axial spacing, the peak RF (RF_max) was positively associated with the SVC drainage fraction (*R* ^2^ = 0.87, n = 6). Given the limited number of configurations and the narrow range of SVC drainage fractions examined, this association should be considered exploratory. Second, reducing axial spacing increased the time-averaged RF (RF_ave) from 2.73% in the baseline configuration to 6.48%. Circumferential distribution of the IVC side holes was associated with a lower RF_ave (2.10%–2.34%) and with reduced stagnation volume, without increased WSS exposure or impaired washout.

**Discussion:**

These results suggest that considering circumferential side-hole placement could be a useful and underexamined parameter for DLC design optimization in VV-ECMO within the investigated geometric range.

## 1 Introduction

Acute respiratory failure remains a major clinical challenge in critical care medicine. Among its manifestations, acute respiratory distress syndrome constitutes the most severe form, with mortality rates ranging from 30% to 45% despite the application of optimal conventional therapies (Bellani et al., 2016). For patients who are refractory to mechanical ventilation, veno-venous extracorporeal membrane oxygenation (VV-ECMO) has emerged as a vital life-support intervention (Tonna et al., 2021). VV-ECMO functions by withdrawing venous blood from the patient, directing it through an oxygenator to facilitate gas exchange, and returning the oxygenated blood to the venous circulation, thereby temporarily substituting pulmonary function. Traditionally, VV-ECMO is implemented using two single-lumen cannulas inserted at distinct venous access sites for drainage and infusion purposes. The advent of dual-lumen cannulas (DLCs), which consolidate both drainage and infusion capabilities into a single device, has enabled single-site cannulation. This innovation reduces the risk of insertion-site infections and enhances patient mobility (Tignanelli et al., 2019; Tipograf et al., 2020; Bazan et al., 2021), leading to a marked increase in the clinical adoption of DLCs over the past decade.

Despite these operational benefits, DLCs present hemodynamic challenges attributable to the complex internal flow patterns generated within the confined geometry of the right atrium (RA). The most clinically significant complication is recirculation, wherein oxygenated blood delivered via the infusion port is immediately re-aspirated into the drainage lumen without passing through the systemic or pulmonary circulations. This phenomenon reduces the effective gas-exchange capacity of the ECMO circuit and may impair patient oxygenation (Conrad and Wang, 2021; Gehron et al., 2023). Furthermore, regions characterized by non-physiological wall shear stress (WSS) and low-velocity stagnation in and around the cannula are implicated in platelet activation, hemolysis, and thrombus formation, thereby contributing to bleeding and thrombotic complications. Consequently, optimizing the hemodynamic design of DLC geometry is imperative to improve clinical outcomes.

Computational fluid dynamics (CFD) has become the primary methodology for assessing the hemodynamic performance of DLC, given the inherent limitations of clinical imaging and *in vitro* experiments in resolving complex internal flow fields. Early numerical and experimental investigations by De Bartolo et al. (2011) on a first-generation DLC identified regions of elevated shear stress and recirculation fraction (RF) that contribute to blood damage. Azimi et al. (2022) systematically examined the influence of side-hole number, diameter, and spacing on hemodynamic parameters using an Avalon 27 Fr DLC model, demonstrating that optimized side-hole configurations effectively reduce stagnation volumes while maintaining low RF. Parker et al. (2023) compared the hemodynamic performance of two commercial DLC (Avalon and Crescent) across multiple axial positions, reporting that substantial axial displacements significantly increase RF, particularly at elevated ECMO flow rates. Wong et al. (2024) further revealed that even minor DLC displacements (5–15 mm) within the RA alter local flow structures, elevate WSS, and increase turbulent kinetic energy. More recently, comparative CFD analyses of various VV-ECMO cannulation configurations have concurrently quantified RF, hemolysis, and thrombosis risk, highlighting the expanding role of CFD in integrated hemodynamic and blood-damage assessments of ECMO cannulas (Xi et al., 2025). Additional investigations focusing on neonatal and pediatric DLC designs have elucidated the impact of patient-specific atrial anatomy on jet impingement and drainage efficiency (Muhammad et al., 2018; Sawka et al., 2023; Ahmad et al., 2023). However, these findings are not directly transferable to adult cannulation due to significant differences in vessel dimensions, cardiac output, and cannula geometry.

Although these studies have provided valuable insights into DLC hemodynamics, several knowledge gaps remain. Most existing parametric investigations have primarily focused on the number and diameter of side holes or the overall positioning of the cannula. However, the independent effects of the axial spacing between the superior vena cava (SVC) drainage holes, inferior vena cava (IVC) drainage holes, and the infusion port (denoted as H_1_ and H_2_) have not been systematically examined. This spacing critically determines the spatial separation between the return jet and drainage suction zones, thereby influencing recirculation behavior. Additionally, the circumferential and axial configurations of the side holes affect the drainage ratio from the SVC to the IVC, subsequently impacting the flow field structure and WSS distribution. To date, no studies have investigated how variations in side hole arrangement alter these hemodynamic outcomes.

This study aims to conduct numerical simulations of hemodynamic behavior under pulsatile venous inflow conditions with constant ECMO flow, employing non-Newtonian blood properties and transient CFD methodologies. By assessing parameters such as RF, WSS, washout efficiency, and regions of stagnation, this research investigates the relationships between geometric design parameters and hemodynamic performance within physiological pulsatile vena cava flow.

## 2 Materials and methods

### 2.1 Geometry and parameterization

To evaluate the hemodynamic performance of DLC under VV-ECMO conditions, a simplified cardiovascular model was employed as the three-dimensional computational domain. This model complies with the ISO 18193:2021 industry standard and incorporates idealized geometries of the IVC, SVC, RA, and tricuspid valve (TV) (ISO, 18193:2021, 2026). The right ventricle and pulmonary artery were not explicitly modeled. As shown in Figure 1, detailed geometric dimensions are provided in Supplementary Table S1. Consistent with previous numerical studies on ECMO cannulation, the DLC was assumed to be positioned concentrically within the venous lumen to avoid interference effects caused by deviations from the central axis (Azimi et al., 2022; Parker et al., 2023; Wong et al., 2024).

**FIGURE 1:**
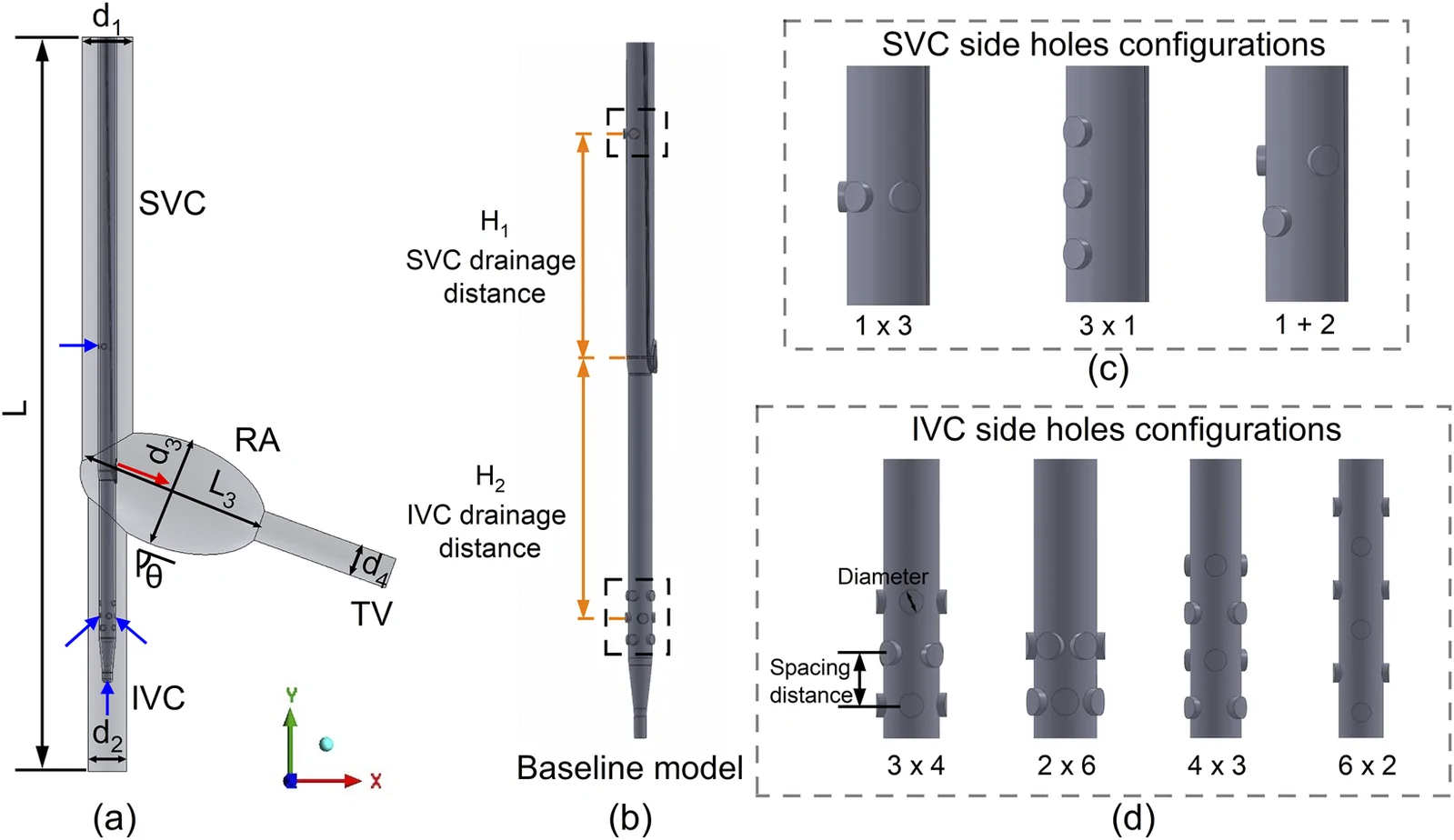
Geometric models and parameter definitions **(a)** Idealized cardiovascular domain built per ISO 18193:2021; full dimensions are given in Supplementary Table S1; **(b)** Baseline DLC model and **(c) (d)** Side hole parameterized configuration. $n_{1}\times n_{2}$ denotes rows × holes per row.

The benchmark DLC model (Model M_1_) is based on the Medtronic Crescent 26Fr catheter design. The drainage cannula features 15 side holes, each with a diameter of 2.5 mm; three are positioned in the SVC and 12 in the IVC. Two geometric parameters are defined to describe the axial positions between the drainage side holes and the infusion port: (1) H_1_, representing the axial distance between the center of the side hole distribution at the SVC drainage port and the center of the infusion hole; and (2) H_2_, representing the axial distance between the center of the side hole distribution at the IVC drainage port and the center of the infusion port. In the baseline configuration (M_1_), H_1_ and H_2_ are set to 50 mm and 60 mm, respectively. Supplementary Table S2 provides detailed side-hole parameters for each configuration, including circumferential angular pitch and axial row spacing.

To systematically evaluate the influence of side-hole distribution on hemodynamic performance, eight representative configuration models were developed. All configurations were derived from the baseline model M_1_, with only one parameter modified at a time. Three research groups were established: (i) the axial spacing group, in which H_1_ and H_2_ were varied simultaneously (M_2_: 30/40 mm; M_3_: 70/80 mm); (ii) the SVC configuration group, where the arrangement of the 3 SVC side holes was altered (M_4_: axial arrangement “ 3×1 ”; M_5_: mixed arrangement “ 1+2 ”), while maintaining H_1_ and H_2_ at 50/60 mm; and (iii) the IVC arrangement group, in which the circumferential arrangement of the 12 IVC side holes was modified (M_6_: “ 2×6 ”; M_7_: “ 4×3 ”; M_8_: “ 6×2 ”), with H_1_ and H_2_ held constant at 50/60 mm. A summary of all configurations is provided in Table 1.

**TABLE 1:** DLC design configurations.

Group	Model	H_1_/H_2_(mm)	SVC side hole arrangement	IVC side hole arrangement
Baseline	M_1_	50/60	3 holes, radial distribution (1 ×3 )	12 holes 3×4
Axial spacing	M_2_	30/40	Same as M_1_	Same as M_1_
	M_3_	70/80	Same as M_1_	Same as M_1_
SVC configuration	M_4_	Same as M_1_	3 holes, axial distribution ( 3×1 )	Same as M_1_
	M_5_	Same as M_1_	3 holes, mixed distribution ( 1+2 )	Same as M_1_
IVC configuration	M_6_	Same as M_1_	Same as M_1_	12 holes 2×6
	M_7_	Same as M_1_	Same as M_1_	12 holes 4×3
	M_8_	Same as M_1_	Same as M_1_	12 holes 6×2

### 2.2 Mesh generation and numerical methods

The computational domain was discretized using unstructured polyhedral meshes. Local mesh refinement was applied in specific regions, including the SVC and IVC drainage side holes and the infusion outlets. The target cell size was set at 0.6 mm, as determined by a mesh independence study (see Supplementary Material). Seven inflation layers, each 0.02 mm thick, were applied along the vena cava and the DLC surface. The final mesh comprised of approximately 3.1 million cells (Supplementary Section 3; Supplementary Table S3).

All simulations were conducted using ANSYS Fluent software (ANSYS Inc., United States of America). Blood was modeled as a homogeneous, incompressible, non-Newtonian fluid with a density of ρ = 1050 kg/m^3^. The Carreau model was employed to characterize the non-Newtonian viscosity, as shown in Equation 1 (Johnston et al., 2004):

$$\mu =\mu_{\infty }+\left(\mu_{0}‐\mu_{\infty }\right){\left[1+{\left(\lambda \dot{\gamma }\right)}^{2}\right]}^{\left(n‐1\right)/2}$$ (1)

where $\mu_{0}=0.056$ Pa·s, $\mu_{\infty }=0.00345$ Pa·s, λ=3.313 s, n=0.3568 (Johnston et al., 2004). The k-ω SST turbulence model was used, consistent with previous studies of DLCs (Azimi et al., 2022; Wong et al., 2024). Local Reynolds numbers based on cycle-averaged quantities (Re*) were approximately 7200 at the infusion outlet, 1730 at the SVC drainage side holes, and 440 at the IVC drainage side holes. These values indicate the coexistence of a higher Re* infusion jet and low Re* drainage flows. To evaluate the sensitivity of turbulence modeling, simulations using both laminar and transition-SST models were conducted for the baseline configuration M_1_. Detailed Re* calculations and flow-model sensitivity results are available in Supplementary Section 5 and Supplementary Tables S5, S6. A cardiac cycle lasting 0.8 s, corresponding to a heart rate of 75 beats per minute (bpm), was simulated with a time step of $2.5\times 10^{‐3}$ seconds. Time step independence was assessed for the baseline configuration M_1_ using additional time steps of $1.25\times 10^{‐3}$ and $5\times 10^{‐3}$ seconds. Detailed methods and results are provided in Supplementary Section 4 and Supplementary Table S4.

Pulsatile physiological boundary conditions, based on volume flow waveform data reported by Mohiaddin et al. for healthy adults (Mohiaddin et al., 1990), were applied at the entrances of the SVC and IVC. The heart rate was maintained at 75 bpm, as illustrated in Figure 2. A constant ECMO flow of 4 L/min was maintained at both the infusion inlet and drainage outlet, representing a reference adult VV-ECMO support condition. Additionally, a flow-rate sensitivity analysis was conducted for the baseline configuration (M_1_) at three and 5 L/min, with all other model parameters remaining unchanged. Further details can be found in Supplementary Section 6. A pressure outlet boundary condition of 0 Pa was applied at the TV. All walls were defined as no-slip surfaces. Convergence was achieved when the residuals of the governing equations fell below 10^–5^. A second-order spatial discretization scheme was employed. To mitigate the influence of initial transient effects, three cardiac cycles were simulated initially to establish a stable flow field. Subsequently, user-defined scalars (UDS) were activated to quantify washout and RF. Cycles 1–3 established a periodic flow field. The recirculation and washout scalars were initialized at the start of cycle 4. The flow distribution was time-averaged over cycle 8, while washout was monitored throughout cycles four to 8, corresponding to a duration of 4.0 s.

**FIGURE 2:**
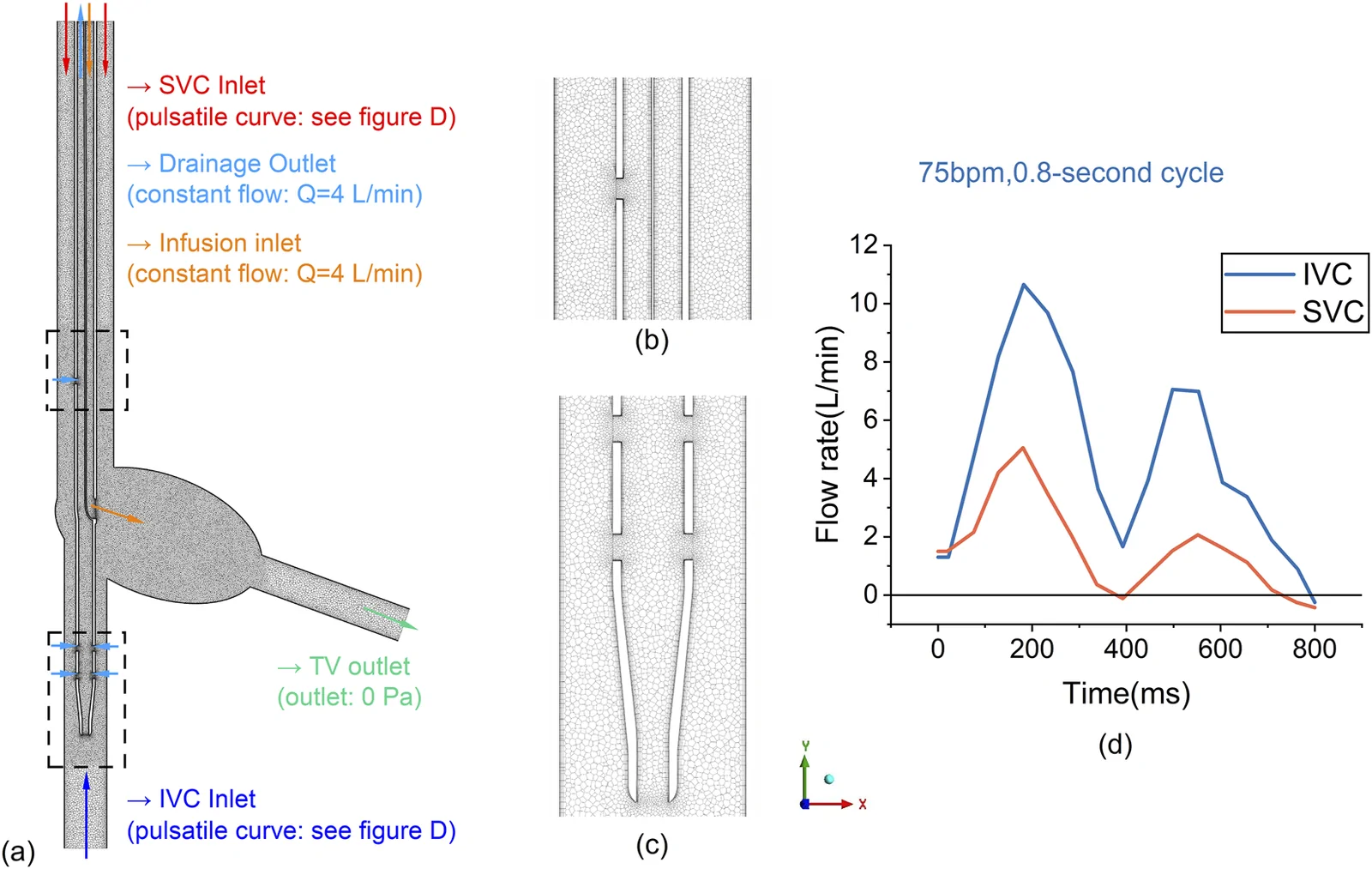
Mesh and boundary conditions. **(a)** Unstructured polyhedral mesh of the full domain. Local refinement at the **(b)** SVC and **(c)** IVC drainage side holes and the infusion outlet. **(d)** Pulsatile SVC and IVC inlet flow waveforms applied over one 0.8 s cardiac cycle (75 bpm).

### 2.3 Performance metrics

During the eighth cardiac cycle, mass flow rates through the SVC side hole, IVC side hole, and IVC end hole were calculated. The flow distribution through the side holes was quantified using surface integrals over the internal cross-sections at the center of each hole. Flow rates within one cycle were averaged for statistical comparison. Results were expressed as a percentage of the total ECMO drainage flow (4 L/min).

RF was defined as the proportion of oxygenated blood that is re-aspirated through the drainage lumen. Throughout the domain, the UDS was initialized to zero, with a value of one assigned exclusively to the oxygenated blood entering through the infusion port. The scalar transport equation was solved with a diffusion coefficient of zero. The volume fraction of the scalar with values greater than zero within the drainage lumen was monitored, representing the RF (%) (Azimi et al., 2022).

Non-physiological WSS and low-velocity retention zones are closely associated with platelet activation and thrombosis, thereby increasing the risk of clinical complications (Casa et al., 2015). Exposure to non-physiological WSS levels reaching 125 Pa for durations exceeding 0.5 s has been shown to sufficiently trigger platelet activation, leading to thrombosis (Chen et al., 2015; Chen et al., 2020). Furthermore, elevated WSS can cause the detachment of certain platelet receptors, compromising platelet adhesion, aggregation, and hemostatic function, which in turn increases the risk of bleeding (Chen et al., 2015). Therefore, it is crucial to monitor both the average WSS and the proportion of the total surface area affected on the walls of the vena cava and DLC to effectively evaluate the potential risk of thrombosis and bleeding (Azimi et al., 2022).

The stagnation region, defined as the region where velocity and strain rate fall below 0.001 m/s and 100 s ^−1^, respectively, represents areas where clotting factors accumulate under low shear conditions (Fraser et al., 2010; Liao et al., 2016). Washout efficiency was defined as the fraction of the domain volume in which the old-blood scalar had decayed to zero, tracked over time to identify regions prone to prolonged blood retention. At the onset of the evaluation phase (the fourth cardiac cycle), a second UDS was initialized to one (representing old blood) throughout the entire domain, while all inlet boundaries were assigned a value of zero (representing newly flowing fresh blood) (Liao et al., 2016). The scalar was transported via a convection-diffusion equation with a zero-diffusion coefficient. This metric was initialized at the fourth cycle after flow stabilization during the initial three cycles and was continuously monitored over the subsequent five cardiac cycles.

### 2.4 Statistical analysis

Statistical analyses were conducted using Origin 2024 (OriginLab Corporation, Northampton, MA, United States of America). Linear regression was employed to evaluate the relationship between the SVC drainage fraction and peak RF (RF_max) across six configurations with identical axial spacing (M_1_, M_4_-M_8_). The regression equation, coefficient of determination (*R* ^2^), p value, and 95% confidence interval of the fit were reported. Due to the limited number of configurations, the regression results were interpreted descriptively.

## 3 Results

### 3.1 Flow field performance

The baseline model (M_1_), used to characterize the overall flow dynamics within the vena cava and RA, is shown in Figure 3. In the longitudinal cross-section (Figure 3a), the infusion jet originates from the infusion lumen and extends toward the TV in the RA, while the drainage flow is drawn into the side holes of the SVC and IVC. A recirculation zone is observed adjacent to the drainage holes, where some of the infused flow is entrained back into the cannula.

**FIGURE 3:**
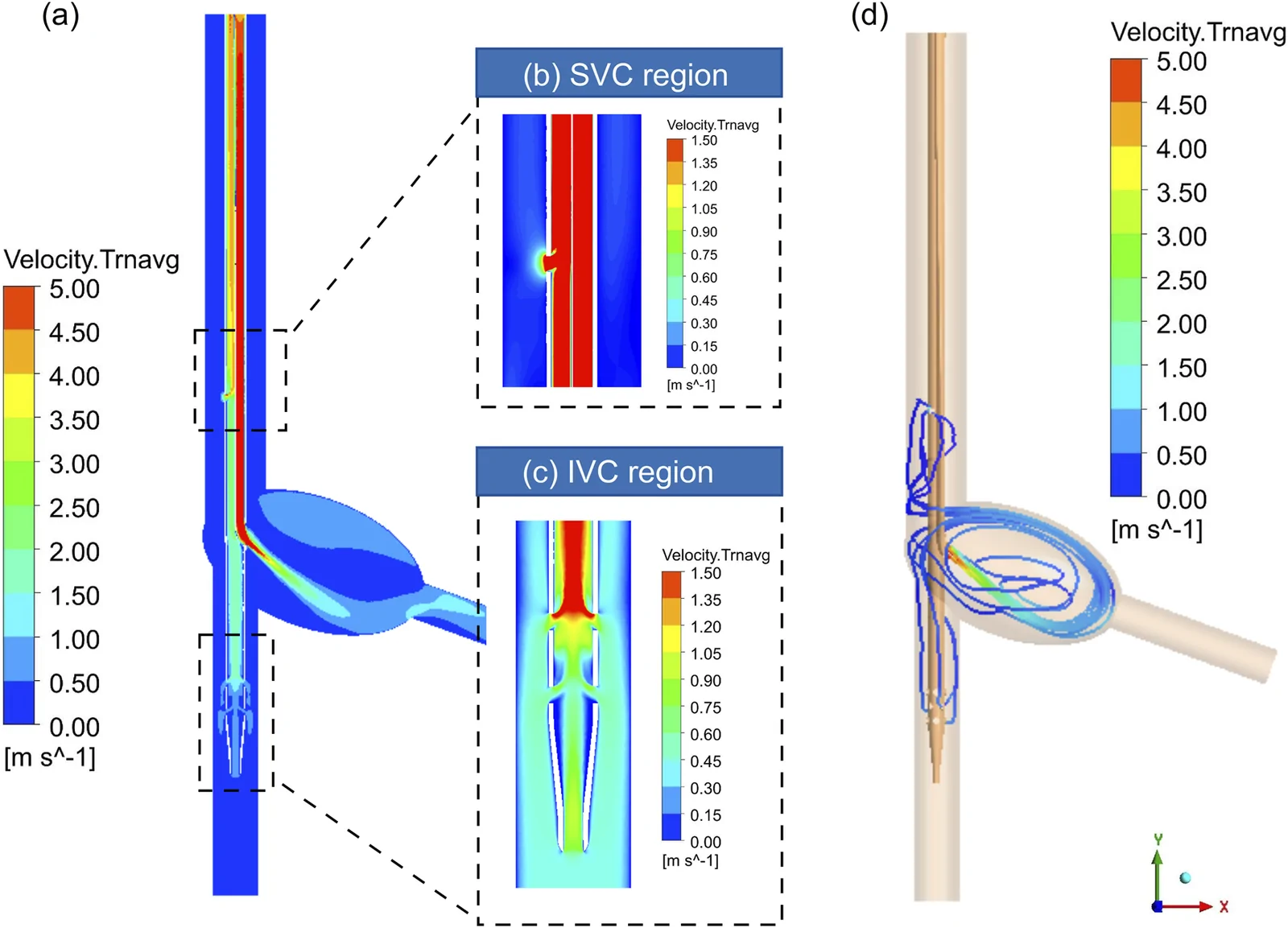
Baseline flow field **(a)** Overall velocity field on the longitudinal mid-plane through the cannula, SVC, IVC and RA. **(b)** Magnified view of the SVC side-hole region. **(c)** Magnified view of the IVC side-hole region. **(d)** Streamlines released from the infusion outlet, colored by velocity magnitude.

A detailed view of the SVC region (Figure 3b) demonstrates that blood flow converges toward the side holes, with velocity increasing at the entry points of these openings. The corresponding magnified view of the IVC region (Figure 3c) shows that the velocity distribution across the 12 IVC side holes is more uniform than in the SVC region. This uniformity is attributed to the larger total side hole area and the reduced flow rate per individual hole.

Streamlines originating from the infusion inlet (Figure 3d) illustrate the trajectory of the return jet within the RA. The infusion flow exits along the proximal axis, generating a large-scale circulation vortex within the RA before exiting through the TV. The peripheral edge of the return jet remains close to the SVC drainage side-hole region. This provides a geometric basis for the quantified recirculation behavior described in Section 3.2.

### 3.2 Flow ratio and recirculation behavior

Figure 4a illustrates the drainage flow distribution through the side holes of the SVC and IVC under ECMO support at a flow rate of 4 L/min across all eight configurations. Both the baseline and side hole configuration models demonstrated an approximately equal distribution of drainage between the two regions. The SVC flow rates ranged from 2.00 to 2.14 L/min, corresponding to 50.0%–53.44% of the total flow. Model M_2_ exhibited the highest SVC drainage fraction at 2.14 L/min (53.44%).

**FIGURE 4:**
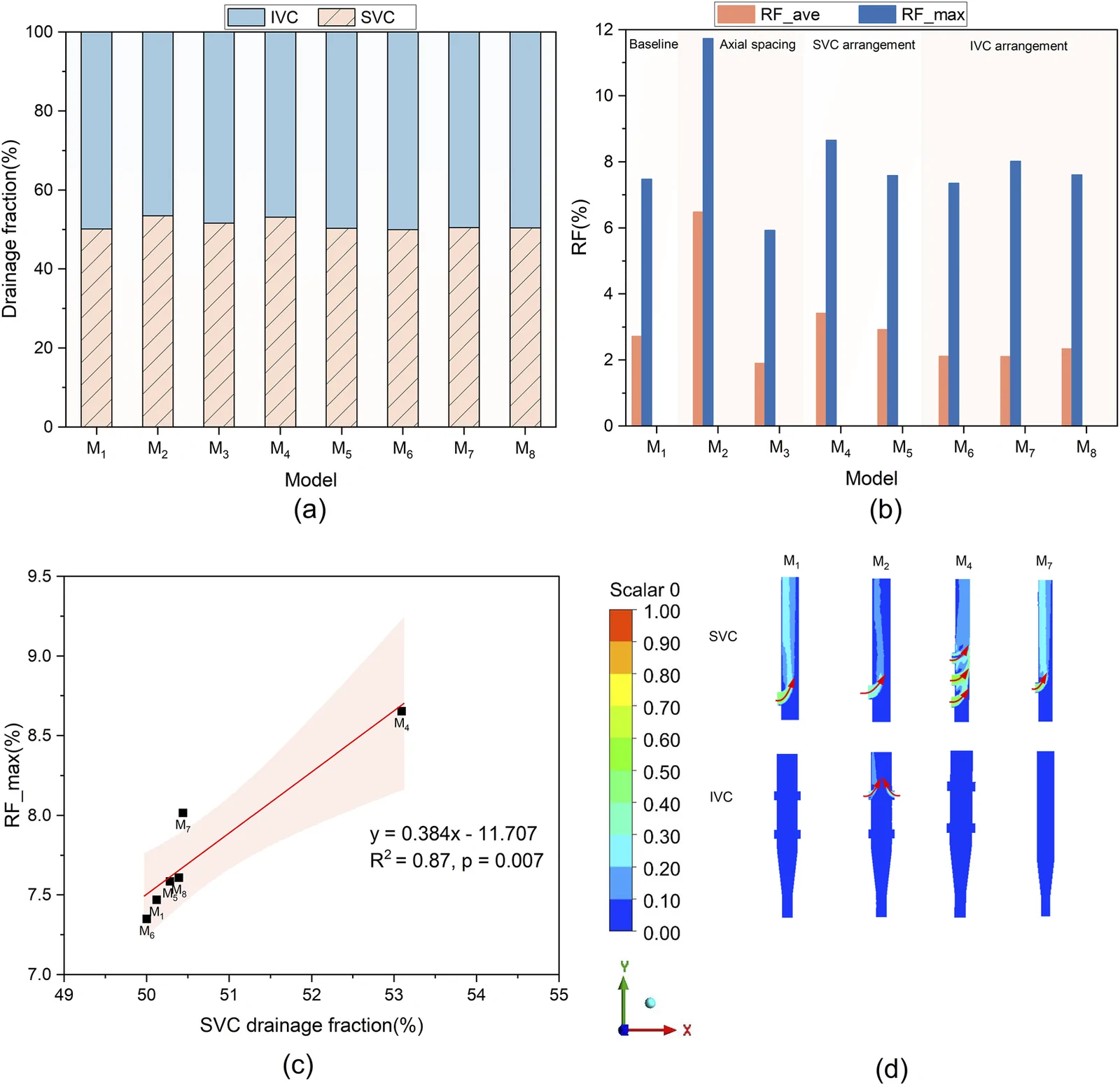
Recirculation behavior for configurations M_1_–M_8_. **(a)** Drainage flow distribution between the SVC and IVC side holes, expressed as a percentage of total ECMO drainage. **(b)** RF_ave and RF_max; **(c)** Relationship between SVC drainage fraction and RF_max. The solid red line depicts the linear regression fit, while the shaded area represents the 95% confidence interval. **(d)** Recirculated-scalar distribution within the SVC (upper) and IVC (lower) drainage lumens for four representative configurations (M_1_, M_2_, M_4_, M_7_).

Figure 4b presents the RF_ave and RF_max for all eight configurations. RF_ave values ranged from 1.90% (model M_3_) to 6.48% (model M_2_), while RF_max values ranged from 5.92% (model M_3_) to 11.73% (model M_2_). The baseline model M_1_ exhibited RF_ave and RF_max values of 2.73% and 7.47%, respectively. The axial-spacing group spanned the full range of recirculation: the short-spacing model M_2_ produced the highest RF of all models (RF_ave 6.48%, RF_max 11.73%), whereas the long-spacing model M_3_ produced the lowest (RF_ave 1.90%, RF_max 5.92%), indicating a monotonic effect of axial spacing. The SVC-arrangement group (models M_4_ and M_5_) showed intermediate RF_ave values of 3.42% and 2.92%, and RF_max values of 8.65% and 7.59%, respectively. The IVC-arrangement group (models M_6_, M_7_, and M_8_) had the lowest RF_ave values at 2.12%, 2.10%, and 2.34%, respectively, with corresponding RF_max values of 7.35%, 8.02%, and 7.61%. Across all models, RF_max exceeded RF_ave by a factor ranging from approximately 1.8–3.8.

Figure 4c illustrates the relationship between RF_max and the SVC drainage fraction for six configurations where axial spacing remained constant (M_1_ and M_4_–M_8_). In these configurations, RF_max generally increased with the SVC drainage fraction (*R* ^2^ = 0.87, p = 0.007, n = 6). M_2_ and M_3_ were not included in the regression because axial spacing was altered in these configurations. The regression is provided as a descriptive summary of the trend observed across the simulated configurations.

Figure 4d illustrates the distribution of the recirculated scalar within the drainage lumen for four representative configurations (M_1_, M_2_, M_4_, M_7_), with the SVC (upper) and IVC (lower) drainage portions shown separately. In the baseline configuration (model M_1_), scalar values of approximately 0.2 were uniformly distributed along the entire SVC drainage lumen. A localized region of higher concentration (approximately 0.4) was situated immediately above the SVC side holes at the inlet. The IVC drainage lumen remained free of scalar contamination. Model M_2_, characterized by the shortest axial spacing, exhibited a distinct pattern. In addition to the SVC drainage contamination similar to that in model M_1_, an elevated scalar concentration of approximately 0.2–0.3 was observed at the upper end of the IVC drainage lumen, indicating direct entrainment of the infusion jet into the IVC side holes. Configuration M_4_ demonstrated the highest localized scalar concentration (approximately 0.5–0.7), confined to a narrow band at the base of the SVC drainage lumen just above the upper SVC side-hole. The upper portion of the lumen exhibited lower scalar values, and the IVC lumen remained uncontaminated. Model M_7_ showed the most confined contamination, with a small region of scalar presence (approximately 0.3) immediately above the SVC side hole entry and near-zero scalar values throughout the rest of both lumens.

Additional flow-rate sensitivity simulations were conducted for M_1_. As the ECMO flow increased from three to 5 L/min, RF_ave rose from 0.57% to 5.02%, with a value of 2.73% at 4 L/min. Meanwhile, RF_max increased from 2.62% to 11.68%. These results are summarized in Supplementary Table S7.

### 3.3 Time-averaged wall shear stress (TAWSS)

Figure 5a shows the percentage of the cannula wall area exceeding each TAWSS threshold. Across all configurations, the majority of the exposed area fell within the lowest range (10 < TAWSS ≤50 Pa). The total cannula wall area with TAWSS greater than 10 Pa ranged from 3.16% (M_3_) to 5.40% (M_2_), with the baseline model M_1_ at 4.09%. The area corresponding to 10 < TAWSS <125 Pa ranged from 3.16% to 5.23%, while the area with TAWSS ≥125 Pa remained below 0.2% in all models, being largest in M_2_ and smallest in M_3_.

**FIGURE 5:**
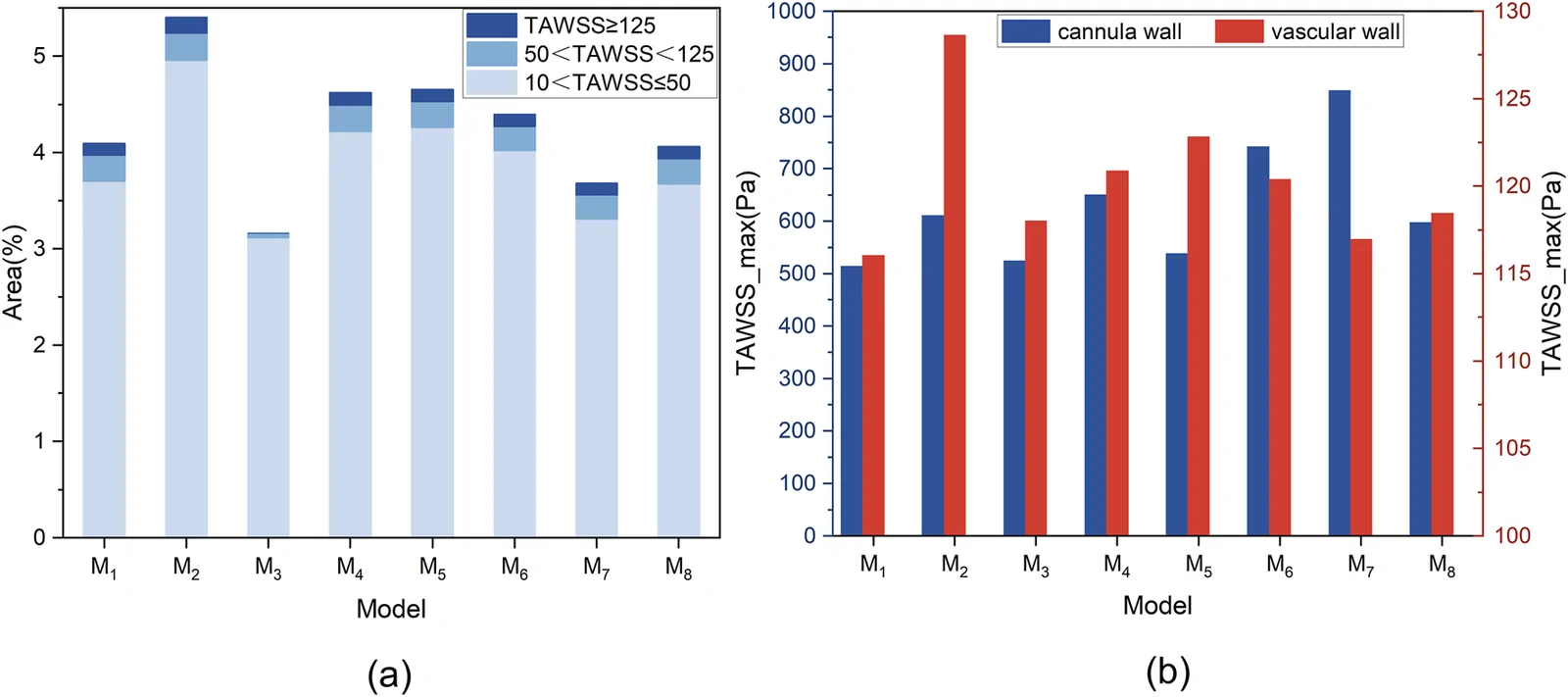
TAWSS on the outer cannula wall and on the vascular wall for M_1_–M_8_. **(a)** Percentage of the cannula-wall surface area exposed to each TAWSS band (10 < TAWSS ≤50 Pa, 50 < TAWSS <125 Pa, and TAWSS ≥125 Pa). **(b)** Maximum cannula-wall TAWSS (left axis) and maximum vascular-wall TAWSS (right axis).

Figure 5b shows the maximum TAWSS on the cannula and vascular walls. The maximum TAWSS observed on the cannula wall was several times greater than the peak value recorded on the vascular wall and exceeded the corresponding mean cannula-wall TAWSS by more than an order of magnitude (11–13 Pa, see Table 2). This finding showed that elevated shear stress was confined to highly localized regions, specifically at the rims of the side holes, rather than being uniformly distributed across the cannula surface. The maximum TAWSS on the cannula wall ranged from 514 Pa in model M_1_ to 850 Pa in model M_7_, with the lowest values observed in M_1_ and the highest in M_7_. In contrast, the maximum TAWSS on the vascular wall exhibited a considerably narrower range, approximately 118 Pa in M_3_ to 123 Pa in M_5_. Notably, although model M_7_ demonstrated the smallest region of elevated TAWSS, it produced the highest localized peak.

**TABLE 2:** Summary of hemodynamic results for all DLC configurations.

Model	SVC drainage fraction (%)	RF_ave (%)	RF_ave vs. M_1_ (%)	Average cannula-wall TAWSS (Pa)	Cannula area TAWSS ≥125 Pa (%)	Average vascular- wall TAWSS (Pa)	Stagnation volume (mm^3^)	Stagnation volume vs. M_1_ (%)	Washout at 4.0 s (%)
M_1_	50.12	2.73	-	11.27	0.12	2.84	63.31	-	96.10
M_2_	53.44	6.48	+137.65	12.55	0.16	3.26	16.66	−73.69	96.46
M_3_	51.55	1.90	−30.28	11.08	0.04	2.96	87.43	+38.10	95.19
M_4_	53.09	3.42	+25.43	11.89	0.13	3.10	98.44	+55.49	96.17
M_5_	50.28	2.92	+7.30	11.48	0.12	3.03	65.55	+3.54	96.05
M_6_	50.00	2.12	−22.24	11.48	0.12	2.98	54.71	−13.58	96.01
M_7_	50.44	2.10	−22.86	11.33	0.12	2.86	46.13	−27.14	96.06
M_8_	50.39	2.34	−14.29	11.36	0.12	2.98	59.43	−6.13	96.04

### 3.4 Stagnation region

The instantaneous stagnation volume at mid-systole, corresponding to peak caval inflow (t = 5.8 s), is presented in Figure 6 for all eight configurations. Across the models, the stagnation volume ranged from 16.66 mm^3^ (M_2_) to 98.44 mm^3^ (M_4_), with the baseline (M_1_) at 63.31 mm^3^. The short-spacing configuration M_2_ (30/40 mm) yielded the lowest stagnation volume among all models, measuring 16.66 mm^3^, which corresponds to a 73.7% reduction relative to M_1_. In contrast, the long-spacing configuration M_3_ (70/80 mm) resulted in an increased stagnation volume of 87.43 mm^3^, representing a 38.1% rise. Both SVC arrangement configurations demonstrated stagnation volumes at or exceeding the baseline: the axial arrangement M_4_ (3 × 1) generated the highest stagnation volume across all configurations, at 98.44 mm^3^ (+55.5%), whereas the mixed arrangement M_5_ (1 + 2) remained near baseline levels, with a stagnation volume of 65.55 mm^3^ (+3.5%). In contrast, all three IVC arrangement configurations reduced the stagnation volume relative to baseline. M_7_ (4 × 3) achieved the lowest value of the group (46.13 mm^3^, −27.1%), followed by M_6_ (2 × 6, 54.71 mm^3^, −13.6%) and M_8_ (6 × 2, 59.43 mm^3^, −6.1%). Notably, the 4 × 3 IVC layout that produced the lowest RF (Section 3.2) also yielded the smallest stagnation volume within its group, indicating that for this configuration the two metrics improved concurrently rather than being traded off.

**FIGURE 6:**
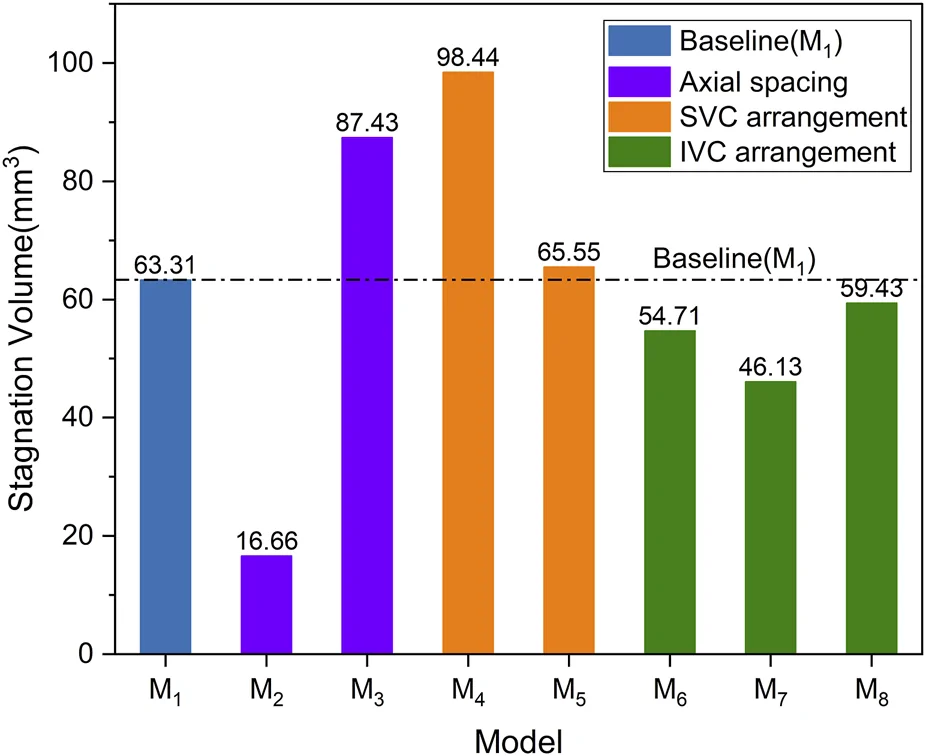
Instantaneous stagnation volume (mm^3^) at mid-systole for M_1_–M_8_.

Figure 7 illustrates the instantaneous spatial distribution of the low-velocity stagnation region at mid-systole, coinciding with peak cava inflow. In all configurations, the stagnation regions were primarily localized around the cannula within the SVC drainage zone and in low-velocity pockets adjacent to the drainage side holes, rather than within the RA. Consistent with the quantitative assessments, the baseline model (M_1_) and the axial SVC arrangement (M_4_) exhibited larger and more continuous low-velocity regions. In contrast, the short-spacing configuration (M_2_) and the circumferential IVC arrangements displayed notably smaller and more fragmented stagnation regions.

**FIGURE 7:**
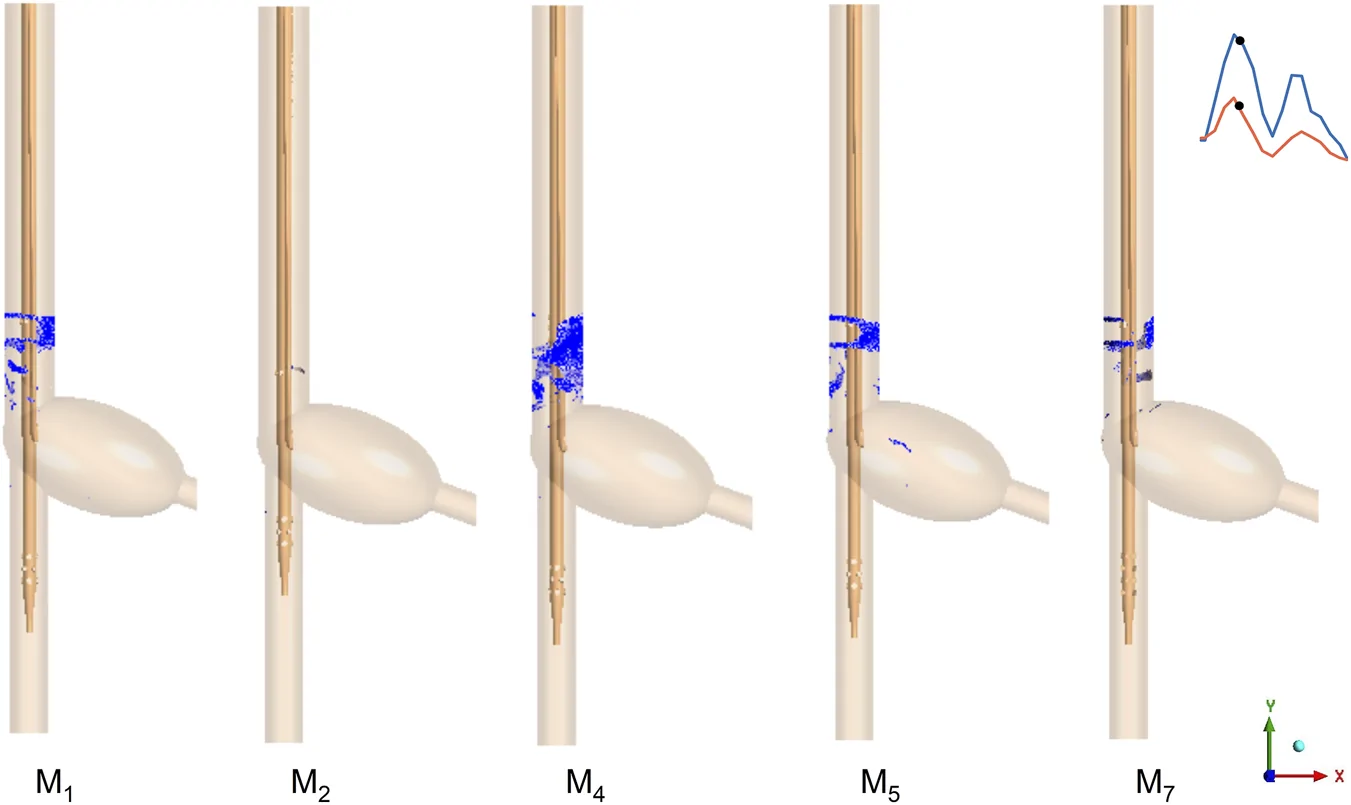
Visualization of stagnation volume at mid-systole.

### 3.5 Washout performance

The washout process was quantified by monitoring the temporal evolution of the calculated cell volume fraction, defined as the scalar concentration decreasing to zero. Over five cardiac cycles (4.0 s), washout efficiency for all configurations is presented in Figure 8. All eight models demonstrated a similar temporal profile, with washout rates increasing rapidly during the initial two cardiac cycles (0–1.6 s) before approaching a steady state. By the end of the evaluation period (t = 4.0 s), washout efficiency exceeded 95% for all configurations, with the maximum inter model difference being less than 1.5%. These findings suggest that the geometric parameters examined did not significantly impact the overall blood-renewal capacity of the RA under the studied conditions. Notably, none of the configurations achieved complete washout within the five cardiac cycles assessed. This is consistent with the 9-s complete washout duration reported by Wong et al. under comparable hemodynamic conditions (Wong et al., 2024).

**FIGURE 8:**
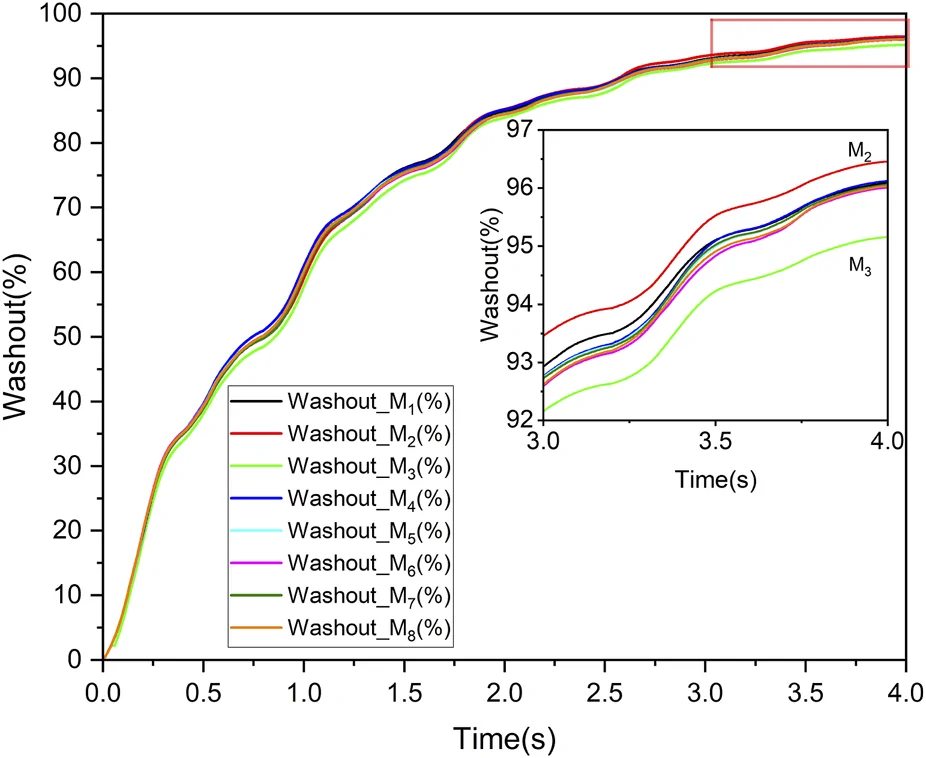
Time evolution of washout for M1–M8 over five cardiac cycles (4.0 s).

Table 2 presents a comprehensive summary of the key hemodynamic parameters across all eight configurations, highlighting trade-offs that are not apparent when considering individual metrics in isolation. Notably, no single configuration demonstrated simultaneous improvement across all evaluated metrics.

## 4 Discussion

Many studies have confirmed the effectiveness of DLC in VV-ECMO therapy. However, complications such as recirculation and elevated WSS persist, thereby limiting clinical outcomes. This study systematically analyzes the effect of the geometric features of side holes on the hemodynamic performance of DLC. Among the design factors evaluated, RF was found to be the parameter most influenced by side-hole design, more so than WSS or washout. Under the simulated conditions, the redistribution of SVC drainage caused by side holes and the axial spacing between the infusion outlet and drainage holes each independently influenced RF, through distinct flow behaviors.

Quantitative comparisons with published data provide indirect support for the plausibility of the predicted RF values. In this study, RF_ave ranged from 1.90% to 6.48%. RF values between 0% and 5.3% have been reported across different side-hole configurations of a 27 Fr DLC (Azimi et al., 2022). At a comparable support flow of 4 L/min, negligible RF was reported in the baseline position, increasing to 3.1% after a small positional displacement of a 27 Fr cannula (Wong et al., 2024). Similarly, RF remained below 7% in correctly positioned adult cannula configurations but increased markedly with cannula malposition (Parker et al., 2023). Thus, the RF values predicted in this study generally fall within the range reported in previous computational studies. However, differences in cannula geometry, positioning, support flow, anatomical models, and RF definition limit direct comparison.

In configurations where axial spacing remained constant (M_1_ and M_4_–M_8_), RF_max was positively associated with the SVC drainage fraction (*R* ^2^ = 0.87, n = 6), consistent with the interpretation that enhanced drainage from the SVC region may increase the likelihood that oxygenated blood returning from the infusion jet is re-aspirated before exiting through the TV. Because the study examined only a few configurations and a limited range of SVC drainage fractions, this relationship should be viewed as exploratory. It is presented as a descriptive summary of the observed trend rather than definitive evidence. The spatial signature of this suction effect is most evident in configuration M_4_. In this setup, the axial 3 × 1 SVC arrangement places the lowest side hole closest to the return jet, causing it to preferentially capture a concentrated stream of recirculated blood (locally 0.5–0.7), while the distal holes contribute minimally. Contamination in M_4_ remained confined to the SVC drainage pathway, and the resulting RF increased moderately above baseline, consistent with its elevated SVC drainage fraction. This highlights the importance of local side-hole positioning in the suction effect.

The additional flow-rate comparison for M_1_ showed that RF_ave increased from 0.57% at 3 L/min to 5.02% at 5 L/min, while the SVC drainage fraction decreased slightly from 52.30% to 49.30% (Supplementary Section 6). Therefore, the association between SVC drainage fraction and RF should be limited to geometric comparisons at a fixed total flow of 4 L/min. Under this condition, the SVC drainage fraction reflects geometry-induced redistribution of drainage between the caval regions rather than absolute suction intensity. The 4 L/min condition represents a reference adult full-support condition, whereas clinical VV-ECMO flow is individualized based on patient size, cardiac output, oxygen-delivery requirements, and recirculation.

Axial spacing had an additional effect on RF that was not captured by the association with SVC drainage fraction. Reducing the spacing increased RF_ave to 6.48% in M_2_, whereas increasing it reduced RF_ave to 1.90% in M_3_. Both configurations deviated from the exploratory relationship observed among the fixed-spacing models. Flow-field analysis revealed that reducing H_1_/H_2_ to 30/40 mm positioned the infusion jet within the overlapping influence regions of both the SVC and IVC drainage holes. Unlike the baseline configuration, where recirculation was primarily confined to the SVC drainage lumen, M_2_ demonstrated scalar contamination in both the SVC and IVC lumens. This behavior suggests a spatial-overlap effect in which insufficient separation between infusion and drainage regions promotes dual-path entrainment of oxygenated blood. Greater separation moved the drainage openings farther from the infusion jet, reducing RF. This sensitivity to axial spacing aligns with previous studies showing that axial displacement and small positional changes can alter recirculation and local flow structures around DLC (Parker et al., 2023; Wong et al., 2024). The present results further suggest that, under the simulated conditions, spacing between the infusion outlet and drainage holes influenced RF even when the overall cannula position remained unchanged.

Among all configurations investigated, the circumferential distribution of IVC side holes (M_6_–M_8_) reduced RF relative to the baseline (RF_ave 2.10%–2.34% versus 2.73% for M_1_). This reduction likely reflects a more even distribution of the drainage load among holes around the cannula circumference, which lowers the local suction intensity at any individual hole and thereby weakens preferential capture of the infusion jet. Importantly, all three arrangements produced similar reductions in RF (differing by less than 0.3%), suggesting that the beneficial effect is associated with the distribution strategy itself rather than a specific hole pattern. Unlike increased axial spacing, these configurations reduced RF without increasing stagnation volume or impairing washout, although the accompanying increase in focal peak shear should also be considered.

WSS exhibited relatively limited sensitivity to the side-hole arrangement when evaluated using global exposure metrics. The mean cannula-wall TAWSS remained within a narrow range of approximately 11–13 Pa across all configurations, while the surface area exposed to TAWSS ≥125 Pa remained below 0.2%. In contrast, the peak TAWSS varied markedly, from 514 Pa in M_1_ to 850 Pa in M_7_. These values exceed the 125 Pa threshold by about four to seven times and are mostly found near the rims of the drainage side holes. Earlier CFD studies on DLCs have shown localized maximum WSS values ranging roughly from 328 Pa up to over 1300 Pa, influenced by cannula design, flow rate, and placement (Azimi et al., 2022; Parker et al., 2023; Wong et al., 2024). The peak values predicted in this study align with the previously reported broad range. These localized increases likely result from flow acceleration and disturbances as blood enters the side holes. Notably, M_7_ decreased RF relative to the baseline but resulted in the highest maximum TAWSS, suggesting that better recirculation performance does not always mean lower focal shear. Although these peaks are limited to small surface areas, they should be evaluated together with overall exposure metrics when refining side-hole geometry.

Washout performance was largely unaffected by the side-hole configuration. All models achieved washout efficiencies exceeding 95% within five cardiac cycles, with differences of less than 1.5% between configurations. These results suggest that blood-renewal capacity within the RA is primarily governed by the overall ECMO flow rate and chamber geometry rather than by the local side-hole arrangement. Similarly, although stagnation volume varied appreciably across configurations, it was not strongly correlated with RF. This lack of a consistent association indicates that RF and stagnation volume characterize different aspects of hemodynamic performance and should be interpreted alongside washout efficiency.

From an engineering design perspective, the present findings support a multi-objective approach for future DLC optimization. Increasing axial separation lowered RF but led to higher stagnation volume and reduced washout efficiency, highlighting a trade-off rather than a universally beneficial change. Therefore, candidate geometries should be assessed collectively through RF, stagnation volume, washout efficiency, and WSS, rather than being chosen solely based on RF.

Several limitations of this study warrant acknowledgment. First, the cardiovascular geometric model used was an idealized representation based on the ISO 18193 standard, rather than being derived from individual patient anatomy. Variations in venous lumen diameter, RA volume, and the orientation of the TV may significantly affect the spatial relationship between the inflow and drainage regions, thereby altering the quantitative thresholds reported herein. Second, the vessel walls and cannula were assumed to be rigid and non-deformable, neglecting fluid-structure interactions. Third, the parametric comparison of side-hole configurations was conducted at a single ECMO flow rate of 4 L/min. Although the baseline configuration was also simulated at three and 5 L/min, the relative impact of the geometric factors examined here may vary across configurations at other flow rates. Fourth, the numerical model lacked validation through specific *in vitro* experiments. While the predicted RF and WSS values generally matched the ranges reported in earlier computational studies, direct experiments such as particle image velocimetry would enhance confidence in these quantitative results. Further parametric studies are necessary to investigate these effects.

## Data Availability

The original contributions presented in the study are included in the article/Supplementary Material, further inquiries can be directed to the corresponding author.
