# Supplementary material for: Influence of side-hole configuration on recirculation in dual-lumen cannulas for veno-venous extracorporeal membrane oxygenation: a parametric computational fluid dynamics study
Source: Front Bioeng Biotechnol. 2026 Sep 18;14:1911749. doi: 10.3389/fbioe.2026.1911749 (PMC13631101; doi:10.3389/fbioe.2026.1911749)
Supplement: Supplementary file 1 [file Table1.docx]

Supplementary Material

# 1 Supplementary Table 1

**Table S1.** Geometric parameters of the idealized cardiovascular model (ISO 18193:2021)

| **Symbol** | **Description** | **Value** |
| --- | --- | --- |
| D | The outer diameter of the cannula | 8.5mm |
| d_1_ | 2.65*D | 22.53mm |
| d_2_ | 2*D | 17.00mm |
| d_3_ | 5.63*D | 47.86mm |
| d_4_ | 1.6*D | 13.60mm |
| L_1_ | 22.5*D | 191.25mm |
| L_2_ | 15.6*D | 132.60mm |
| L_3_ | 10*D | 85.00mm |
| L | L_1_+L_2_ | 323.85mm |
| θ | Right ventricular Angle | 70° |

# 2 Detailed geometry of the side-hole configurations

The geometric parameters of the eight DLC configurations are summarized in Table S2. The definitions of H_1_ and H_2_ and visual representations of the SVC and IVC side-hole arrangements are provided in Figure 1 of the main manuscript.

**Table S2.** Detailed side-hole geometry of the eight DLC configurations

| **Model** | **SVC**  **holes** | **IVC**  **holes** | **Hole diameter (mm)** | **H_1_ (mm)** | **H_2_ (mm)** | **SVC arrangement (axial rows× holes per row)** | **IVC arrangement (axial rows× holes per row)** | **SVC circumferential angular pitch (**°) | **IVC circumferential angular pitch (**°) | **Axial row spacing (mm)** |
| --- | --- | --- | --- | --- | --- | --- | --- | --- | --- | --- |
| M_1_ | 3 | 12 | 2.5 | 50 | 60 | 1×3 | 3×4 | 67.5 | 90 | 5.5 |
| M_2_ | 3 | 12 | 2.5 | 30 | 40 | 1×3 | 3×4 | 67.5 | 90 | 5.5 |
| M_3_ | 3 | 12 | 2.5 | 70 | 80 | 1×3 | 3×4 | 67.5 | 90 | 5.5 |
| M_4_ | 3 | 12 | 2.5 | 50 | 60 | 3×1 | 3×4 | N/A | 90 | 5.5 |
| M_5_ | 3 | 12 | 2.5 | 50 | 60 | 1+2 (mixed) | 3×4 | 135 (two-hole row) | 90 | 5.5 |
| M_6_ | 3 | 12 | 2.5 | 50 | 60 | 1×3 | 2×6 | 67.5 | 60 | 5.5 |
| M_7_ | 3 | 12 | 2.5 | 50 | 60 | 1×3 | 4×3 | 67.5 | 120 | 5.5 |
| M_8_ | 3 | 12 | 2.5 | 50 | 60 | 1×3 | 6×2 | 67.5 | 180 | 5.5 |

Note: n₁ × n₂ denotes the number of axial rows multiplied by the number of circumferential holes per row. The circumferential angular pitch represents the center-to-center angle between adjacent holes within the same axial row. For M_4_, this parameter was not applicable because each row contained only one hole.

# 3 Mesh independence study

A transient mesh independence study was performed using four unstructured polyhedral meshes containing approximately 0.5 million, 1.5 million, 3.1 million and 4.6 million cells. All simulations were performed at an ECMO support flow rate of 4 L/min, with all numerical settings kept constant except for the mesh resolution. The parameters evaluated included RF_ave, RF_max, average cannula-wall TAWSS, average vascular-wall TAWSS, stagnation volume, washout at 4.0 s, and maximum y+, using the same definitions and evaluation intervals as in the primary simulations.

RF_ave increased from 2.30% on the coarsest mesh to 2.54%, 2.73% and 2.75% with successive mesh refinement. The difference between the two finest meshes was only 0.02 percentage points, corresponding to a relative difference of 0.73% from the finest-mesh result. RF_max ranged from 6.90% to 8.08% across all meshes, with a difference of 7.55% observed between the 3.1 million and 4.6 million cell mesh. Average cannula-wall TAWSS remained between 11.27 and 12.65 Pa, representing a total variation of 12%, while average vascular-wall TAWSS varied by less than 6%. Stagnation volume ranged from 63.31 to 65.80 mm^3^ across the three finest meshes, differing by 3.78% between the 3.1 million and 4.6 million cell mesh. Washout at 4.0 s remained consistent, between 95.37% and 96.15%, with a variation of less than 1%. Near-wall resolution improved steadily with mesh refinement, as the maximum y⁺ on the cannula and vessel walls decreased from 9.8 on the coarsest mesh to 3.67, 1.98 and 1.27 on the 1.5, 3.1 and 4.6 million cell meshes, respectively. Given the close agreement in primary outcomes and the significantly higher computational cost of the finest mesh, the 3.1 million mesh was chosen for subsequent simulations. The maximum and average skewness values were 0.870 and 0.028, respectively, while the minimum and average orthogonal quality values were 0.123 and 0.971. Although the maximum skewness indicated the presence of locally distorted cells, the low average skewness and high average orthogonal quality values demonstrated overall good mesh quality.

**Table S3.** Mesh independence results

| **Case** | **Number**  **(**$\text{×}\text{10}^{\text{6}}$**)** | **RF_ave (%)** | **RF_max (%)** | **Average cannula-wall TAWSS (Pa)** | **Average vascular-wall TAWSS (Pa)** | **Stagnation volume (mm^3^)** | **Washout at 4.0s (%)** | **Maximum Y+** |
| --- | --- | --- | --- | --- | --- | --- | --- | --- |
| 1 | 0.5 | 2.30  (−16.36%) | 7.09  (−12.25%) | 12.65  (+4.98%) | 2.73  (−5.54%) | 64.58  (−1.85%) | 95.37  (−0.81%) | 9.80 |
| 2 | 1.5 | 2.54  (-7.64%) | 6.90  (−14.60%) | 11.89  (−1.33%) | 2.79  (−3.46%) | 63.52  (−3.47%) | 95.79  (−0.37%) | 3.67 |
| 3 | 3.1 | 2.73  (-0.73%) | 7.47  (−7.55%) | 11.27  (−6.47%) | 2.84  (−1.73%) | 63.31  (−3.78%) | 96.10  (−0.05%) | 1.98 |
| 4  (reference) | 4.6 | 2.75 | 8.08 | 12.05 | 2.89 | 65.80 | 96.15 | 1.27 |

Note: Values in parentheses represent relative differences from the finest-mesh solution containing 4.6 million cells. $\text{Relative difference (\%) = }\text{(}\text{X}_{\text{mesh}}\text{ - }\text{X}_{\text{finest}}\text{) / }\text{X}_{\text{finest}}\text{ }\text{× 100\%.}$

# 4 Transient time step independence study

Time step sensitivity was assessed using the selected mesh and time steps of $\text{5.0×}\text{10}^{\text{-3}}$, $\text{2.5×}\text{10}^{\text{-3}}$, and $1.\text{25×}\text{10}^{\text{-3}}$s. Differences in scalar outcomes were quantified relative to the smallest time step. Compared to the $1.\text{25×}\text{10}^{\text{-3}}$ s solution, the chosen time step of $\text{2.5×}\text{10}^{\text{-3}}$ s resulted in relative differences of 2.15% in RF_ave, 2.61% in RF_max, 0.88% in average cannula-wall TAWSS, 1.39% in average vascular-wall TAWSS, 7.93% in mid-systolic stagnation volume, and 0.14% in washout at 4.0 s. In contrast, the coarser time step of $\text{5.0×}\text{10}^{\text{-3}}$ s differed from the reference by 22.22% in RF_ave and 11.90% in stagnation volume. Therefore, reducing the time step from $\text{2.5×}\text{10}^{\text{-3}}$ to $1.\text{25×}\text{10}^{\text{-3}}$ s produced only minor changes in RF and WSS outcomes, although stagnation volume was somewhat more sensitive. A time step of $\text{2.5×}\text{10}^{\text{-3}}$ s was consequently selected to balance temporal resolution with computational cost.

**Table S4.** Time-step independence results

| **Time step (**$\text{×}\text{10}^{\text{-3}} \text{s}$**)** | **RF_ave (%)** | **RF****_max (%)** | **Average cannula-wall TAWSS (Pa)** | **Average vascular-wall TAWSS (Pa)** | **Stagnation volume (mm^3^)** | **Washout at 4.0s (%)** |
| --- | --- | --- | --- | --- | --- | --- |
| 5 | 2.17  (-22.22%) | 7.04  (−8.21%) | 11.27  (-0.88%) | 2.87  (-0.35%) | 60.58  (-11.90%) | 96.00  (+0.03%) |
| 2.5 | 2.73  (-2.15%) | 7.47  (-2.61%) | 11.27  (-0.88%) | 2.84  (-1.39%) | 63.31  (-7.93%) | 96.10  (+0.14%) |
| 1.25  (reference) | 2.79 | 7.67 | 11.37 | 2.88 | 68.76 | 95.97 |

Note: Values in parentheses represent relative differences from the smallest time step solution. $\text{Relative difference (\%) = (}\text{X}_{\text{time step}}\text{ - }\text{X}_{\text{reference}}\text{) / }\text{X}_{\text{reference}}\text{ }\text{×}\text{ 100\%.}$

# 5 Local Reynolds number assessment and flow model sensitivity analysis

Local Reynolds numbers were evaluated at the infusion outlet and drainage side-hole cross-sections to characterize the flow regimes and inform the selection of an appropriate turbulence model. The Reynolds number (Re*) was calculated as follows:

$\text{Re}\text{*}\text{=}\frac{\text{ρ}\bar{U}\text{D}_{\text{h}}}{\bar{\mu}}$	(S1)

$\text{D}_{\text{h}}\text{=}\frac{\text{4A}}{\text{P}}$	(S2)

where $\text{ρ = 1050 kg/}\text{m}^{\text{3}}$ is the blood density,$\bar{U}$ is the cycle-averaged, area-averaged velocity across the corresponding opening, Dh is the hydraulic diameter, A is the cross-sectional area, P is the wetted perimeter, and $\bar{\mu}$ is the cycle-averaged apparent dynamic viscosity determined using the Carreau model. Velocity and apparent viscosity fields from the final periodic cardiac cycle were time-averaged and then area-averaged over each cross-section. Because the drainage side holes were circular, their hydraulic diameter equaled the hole diameter. As summarized in Table S5, Re* was approximately 7200 at the infusion outlet, 1730 at the SVC drainage side holes, and 440 at the IVC drainage side holes. The infusion jet therefore operated at a substantially higher Re than the drainage flows. The IVC side-hole flow was within a nominally laminar regime, whereas the SVC side-hole flow occurred at a higher but still relatively low Re*. These results indicate that the cannula contained coexisting low Re* drainage flows alongside a higher Re* infusion jet, rather than a uniformly laminar flow field. This observation motivated the flow-model sensitivity analysis described below.

A separate flow-model sensitivity analysis was conducted using the laminar, k–ω SST, and Transition-SST models (Table S6). The Transition-SST model produced results broadly comparable to those from the k-ω SST solution, with relative differences of 6.96% in RF_ave, 4.95% in RF_max, 0.53% in average cannula-wall TAWSS, 6.69% in average vascular-wall TAWSS, 0.60% in stagnation volume, and 0.22% in washout at 4.0 s. In contrast, the laminar solution deviated more substantially. Compared with the k–ω SST solution, it yielded values that were 20.15% lower for RF_ave, 9.37% lower for RF_max, 7.81% lower for average cannula-wall TAWSS, and 10.92% lower for average vascular-wall TAWSS, whereas stagnation volume and washout differed by only +1.99% and +0.81%, respectively. These results indicate that RF and wall shear stress predictions are more sensitive to flow-regime treatment than stagnation volume and washout. The overall agreement between the k–ω SST and Transition-SST solutions support retaining the k–ω SST model for the primary simulations.

**Table S5.** Local Reynolds numbers at the infusion outlet and drainage side holes

| **Location** | **D_h_**  **(mm)** | **Area-averaged velocity**  **(m/s)** | **Local viscosity**  **(Pa s)** | **Re*** |
| --- | --- | --- | --- | --- |
| Infusion outlet | 5.37 | 4.57 | 0.00358 | ≈ 7200 |
| SVC side holes | 2.5 | 2.35 | 0.00356 | ≈ 1730 |
| IVC side holes | 2.5 | 0.62 | 0.00373 | ≈ 440 |

Note: Re* denotes the Reynolds number calculated using the cycle-averaged area-averaged velocity and cycle-averaged apparent viscosity.

**Table S6.** Flow model sensitivity analysis for the baseline configuration

| **Model** | **RF_ave (%)** | **RF_max (%)** | **Average cannula-wall TAWSS (Pa)** | **Average vascular-wall TAWSS (Pa)** | **Stagnation volume (mm^3^)** | **Washout at 4.0s (%)** |
| --- | --- | --- | --- | --- | --- | --- |
| Laminar | 2.18  (-20.15%) | 6.77  (-9.37%) | 10.39  (-7.81%) | 2.53  (-10.92%) | 64.57  (+1.99%) | 96.88  (+0.81%) |
| k-ω SST  (reference) | 2.73 | 7.47 | 11.27 | 2.84 | 63.31 | 96.10 |
| Transition-SST | 2.54  (-6.96%) | 7.84  (+4.95%) | 11.33  (+0.53%) | 3.03  (+6.69%) | 63.69  (+0.60%) | 95.89  (-0.22%) |

Note: Values in parentheses represent the relative differences from the k–ω SST solution, calculated as $\left( \text{X}_{\text{model}} \text{-}\text{ }\text{X}_{\text{k-ω SST}} \right)/{\text{X}_{\text{k-ω SST}}\text{ }\text{×100\%}}$.

# 6 Influence of ECMO flow rate on drainage distribution and recirculation

A parametric comparison of side-hole configurations was conducted at a fixed ECMO flow rate of 4 L/min to isolate the effects of geometry. To evaluate the impact of extracorporeal flow, the baseline configuration M_1_ was also simulated at 3 and 5 L/min, covering the typical flow range used for adult respiratory support, while keeping all other parameters constant. RF_ave increased steadily from 0.57% at 3 L/min to 2.73% at 4 L/min and 5.02% at 5 L/min, with RF_max increasing correspondingly from 2.62% to 11.68% (Table S7). The SVC drainage fraction decreased from 52.30% to 49.30%, whereas the absolute SVC drainage flow increased from 1.57 to 2.47 L/min. Therefore, the association between SVC drainage fraction and RF observed in the primary geometric comparison should not be extrapolated across different ECMO flow rates. Because absolute drainage flow and return-jet momentum changed simultaneously, their individual contributions to the increase in RF could not be isolated. The SVC drainage fraction should therefore be interpreted as a descriptor of geometry-induced drainage redistribution under a fixed total flow rather than as a measure of absolute suction intensity across different flow rates.

**Table S7.** Influence of ECMO flow rate on drainage distribution and recirculation in the baseline configuration (M_1_)

| **Flow rate**  **(L/min)** | **SVC drainage flow**  **(L/min)** | **SVC drainage fraction**  **(%)** | **RF_ave**  **(%)** | **RF_max**  **(%)** |
| --- | --- | --- | --- | --- |
| 3 | 1.57 | 52.30 | 0.57 | 2.62 |
| 4 | 2.00 | 50.12 | 2.73 | 7.47 |
| 5 | 2.47 | 49.30 | 5.02 | 11.68 |
